# Review of Visual Measurement Methods for Metal Vaporization Processes in Laser Powder Bed Fusion

**DOI:** 10.3390/mi14071351

**Published:** 2023-06-30

**Authors:** Jiaqi Liu, Bin Wei, Hongjie Chang, Jie Li, Guang Yang

**Affiliations:** 1 College of Mechanical Engineering, Hebei University of Science and Technology, Shijiazhuang 050000, China; ljq15003268055@163.com (J.L.); weibin0@126.com (B.W.); sjzchj@126.com (H.C.); 2 Shijiazhuang Information Engineering Vocational College, Shijiazhuang 050000, China

**Keywords:** laser powder bed fusion, metal evaporation, melt pool, evaporation products, visual measurement

## Abstract

Laser powder bed fusion (LPBF) is of great importance for the visual measurement and analysis of the metallization process, which is the process of solid, liquid, and gas phase transformations of metal powders under high-energy laser irradiation due to the low boiling point/high saturated vapor pressure. Since the evaporation of metals involves the interaction of driving forces such as vapor back pressure, surface tension, and gravity, the movement of the melt pool is not stable. At the same time, it also produces vaporization products such as vapor plumes and sprays, which cause defects such as bubbles, porosity, lack of fusion, inclusions, etc., during the manufacturing process of the parts, affecting the performance and manufacturing quality of the parts. More and more researchers are using imaging technologies, such as high-speed X-ray, high-speed visible light cameras, and high-speed schlieren imaging, to perform noncontact visual measurements and analyses of the melt pool, vapor plume, and spatter during the metal evaporation process, and the results show that the metal evaporation process can be suppressed by optimizing the process parameters and changing the processing atmosphere, thereby reducing part defects and improving part performance and built part quality. This paper reviews the research on metal evaporation mechanisms and visual measurement methods of metal evaporation, then discusses the measures of metal evaporation, and finally summarizes and prospects the future research hotspots of LPBF technology, according to the existing scholars’ research on numerical simulation analysis and visual measurement methods of the metal evaporation process.

## 1. Introduction

**Figure 1:**
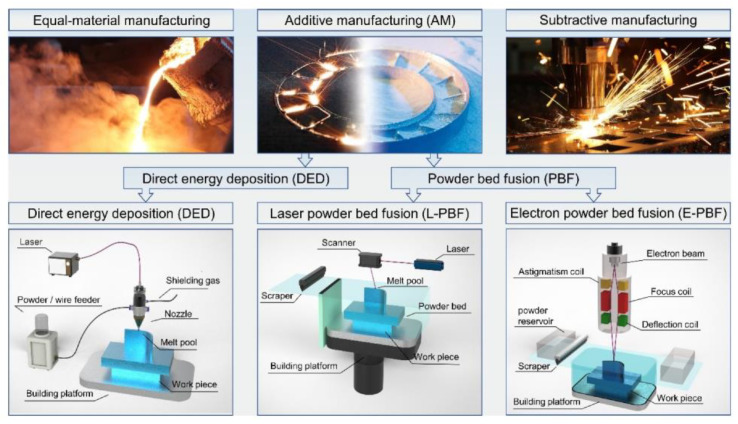
Classification of metal manufacturing processes: Equal-material manufacturing, additive manufacturing, and subtractive manufacturing [23].

**Figure 2:**
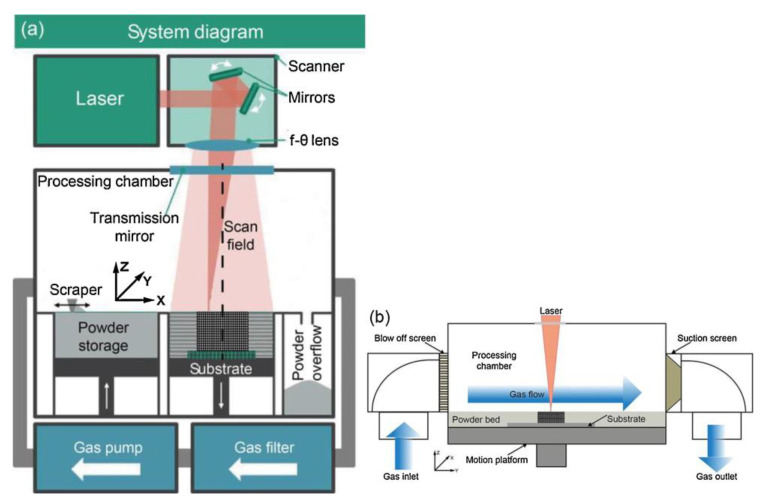
Schematic diagrams of the laser powder bed melting process and the gas circulation: (**a**) the laser powder bed melting process; (**b**) the cross-section of the treatment chamber in the YZ direction [32].

**Figure 3:**
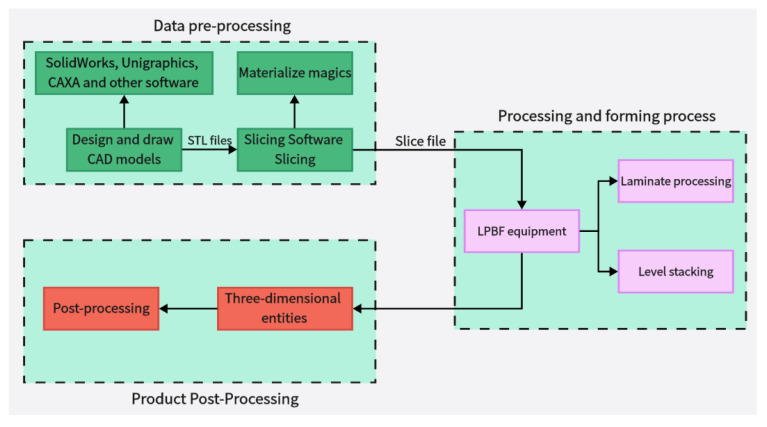
The printing process of LPBF technology.

**Figure 4:**
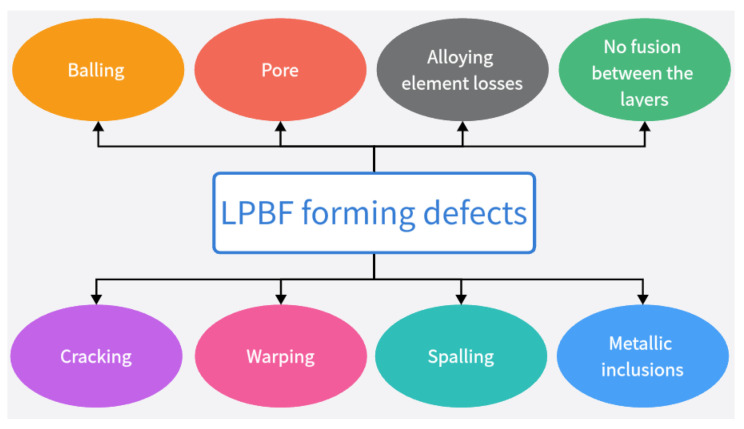
Defects in LPBF.

Additive manufacture (AM) technology (also known as 3D-printing technology) is a new production technology developed in the late 1980s [1,2,3]; the processing principle is the opposite of traditional additive and subtractive production technology [4], with a parts production process using layer-by-layer stacking production process [5]. The technology offers design flexibility, the printing of complex components [6], and lightweight, personalized design [7]; Mg, Cu, Fe, Al, Mo, and other metals and their alloys can be formed [8,9,10,11,12,13,14,15,16,17,18,19,20,21], so it is widely used in the aerospace, energy, biomedical, and automotive industries and other fields of metal parts manufacturing [22]. The metal manufacturing processes mainly include equal-material manufacturing, subtractive manufacturing, and additive manufacturing, as shown in Figure 1 [23]. Metal additive manufacturing is one of the most difficult and advanced additive manufacturing technologies; among metal additive manufacturing technologies, direct energy deposition (DED) [24,25] and powder bed fusion (PBF) [26,27] techniques are the most widely used. Powder bed fusion (PBF) is an additive manufacturing technology used to produce metal parts from metal powder raw materials with two types of input energy: laser and electron [28,29,30]. Among these, LPBF has become a key technology for metal additive manufacturing because of its excellent mechanical properties and high accuracy of formed parts [31]. The LPBF process and gas circulating system is schematically shown in Figure 2 [32]. Figure 2a shows LPBF process equipment, which mainly consists of a laser, scanning mirror, f-θ lens, protecting mirror, scraper, substrate, gas pump, and powder bin. Figure 2b shows the schematic diagram of the circulation of the gas in the forming vessel. This process is also known as selective laser melting (SLM), direct metal laser sintering (DMLS), or laser metal melting (LMF) due to the selective melting of metal powders by a high-energy laser beam on a powder bed according to a designed digital model [33,34]; the printing process of the LPBF technology is shown in Figure 3. At present, LPBF technology has been successfully used to print on Mg, Cu, Fe, Al, Mo, and other metals and their alloys, and the formed metal parts have been widely used in military and civilian applications. LPBF technology has a promising future in aerospace fuel nozzle fabrication [35], automotive engine bay fabrication [36], biomedical bone implant fabrication [37], and more. The use of LPBF technology to produce high-quality, high-performance metal parts has become a sought-after goal with the increasing demand for performance and quality metal parts in various fields. However, LPBF technology is affected by material properties, process parameters, and the external environment, resulting in defects such as spheroidization, porosity, alloy loss, cracking, warping, spalling, incomplete fusion, and inclusions in metal parts, reducing part performance and forming quality [38,39,40,41,42,43]; potential defects in LPBF-produced parts are shown in Figure 4. In order to improve the forming quality of the parts and to reduce the defects in the parts, more and more scholars have begun to study the physical and kinematic processes of the interaction of the laser and the metal powder. In the LPBF process, metal vaporization occurs in addition to the melting of metal powder, and this vaporization has significant effects on the LPBF process and is the key to the quality control of metal parts [44,45].

The LPBF technique is a process of interaction between a high-energy laser and a metal powder, which undergoes changes between the solid, liquid, and gas phases under the irradiation of a high-energy laser, resulting in metal vaporization [46]. Metal vaporization has the following effects: (1) the vaporization of the metal creates vapor recoil pressure above the melt pool, which in turn leads to keyhole cavities in the melt pool, accelerating the flow of liquid in the melt pool while also predisposing the metal to defects such as porosity [47]; (2) metal vaporization can cause alloying elements to burn out and distort the composition of the metal material, affecting the mechanical properties of the part [48]; (3) the metal evaporation process will produce plumes and spatters and other evaporation products, affecting the fluidity of the melt pool. Plumes, spatters, and other evaporation products, on the one hand, will hinder the propagation of high-energy laser radiation, resulting in the metal powder being unable to fully absorb the high-energy laser and the production of an unstable melt pool, destroying the continuity and uniformity of the melt trajectory; meanwhile, sputtering will sputter down to the powder bed, affecting the quality of powder deposition [49]. On the other hand, it will gradually fall on the laser protection mirror, causing optical system damage. Therefore, defects such as porosity, spheroidization, lack of fusion, slagging, etc., are directly related to the vaporization of the metal [50,51,52,53,54], especially for metallic materials such as Mg, Zn, Al, and their alloys, which are prone to vaporization and have important applications. Therefore, it is important to understand the process of laser interaction with metal powders and to make visual measurements of the metal vaporization process [55,56,57,58,59,60]. The laser–metal powder interaction process is a highly dynamic and complex behavior that is a challenge to study. A deeper understanding of the metal evaporation process is lacking [61,62], and visual measurement of it can help us gain a deeper understanding of the physical and kinematic processes behind it, and thus reduce part defects through the optimization of process parameters and control of factors such as the building atmosphere. Recently, it has been found that more and more studies emphasize the effect of metal evaporation [63]. Researchers have used visual measurement methods, such as high-speed X-ray imaging [64,65], high-speed visible camera imaging [66,67] and high-speed schlieren imaging [68,69], to analyze the metal evaporation process and clearly monitor the interaction process between the laser and metal powder. Therefore, considering the future development of LPBF technology applications for additive manufacturing, this paper summarizes the current visual measurement methods for the metal evaporation process, so that more researchers can understand different visual measurement methods and then solve metal evaporation problems. This review paper is organized as follows: Section 2 first explains the mechanism of metal evaporation; Section 3 discusses the visual measurement methods for metal evaporation; Section 4 discusses metal evaporation suppression measures; and Section 5 provides a conclusion.

## 2. Mechanism of Metal Evaporation in the LPBF Process

**Figure 5:**
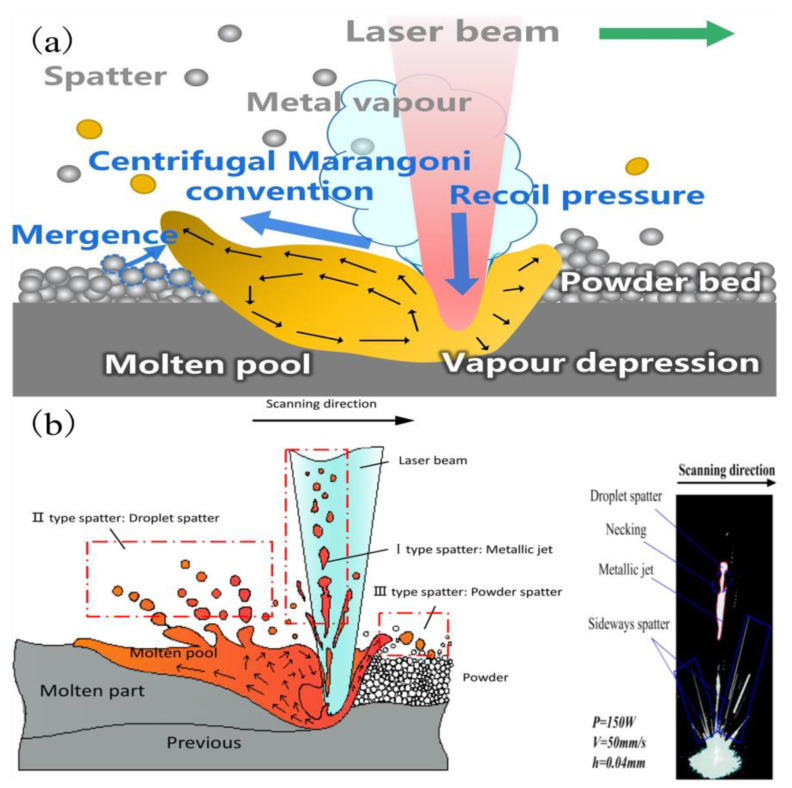
(**a**) Mechanism of posterior protrusion of the melt pool due to vapor recoil pressure and Marangoni convection [53]; (**b**) Formation mechanisms of spatter: three different types of spatters and typical spatter behavior during LPBF [77].

Metal vaporization is an important physical phenomenon during the interaction between laser and metal powder, which is the key to melt pool characteristics and forming quality [70], and by studying the interaction between laser and metal powder, the mechanism of defect generation in LPBF and the factors affecting its forming quality can be revealed. The degree of vaporization varies from one metal material to another due to different metal material characteristics and process conditions [71]. Metal evaporation involves rapidly melting metal powder material by high-energy laser radiation and forming melt puddles as the material temperature reaches the melting point; as the temperature continues to approach the boiling point, the metal vapor suddenly expands into the surrounding air. The vapor expansion creates vapor recoil pressure on the molten surface [72], which increases its penetration depth and creates a gas-filled or plasma-filled depression, often referred to as a keyhole [51]. Porosity defects are caused by keyhole collapse, trapping shielding gas in the melt pool [73] and creating porosity defects, while the high saturation vapor pressure of alloying elements exerts recoil pressure on the melt pool liquid surface [74], causing unstable melt pool flow and droplet splashing, increasing porosity and other defects. At the same time, metal vaporization causes volatile alloying elements to evaporate, resulting in alloy composition segregation, which affects the chemical composition, microstructure, and properties of the part [75]. LPBF is an intense form of laser-induced metal evaporation due to the high energy density of the laser power and the fast-scanning speed, so more severe physical phenomena, such as bursts, may occur in the laser–metal powder interaction during LPBF [76]. In addition, during LPBF, there is significant interaction between the different phases (solid, liquid, and gas), with gas–solid and gas–liquid interactions resulting in vaporization products such as plumes and sputtering. The following processes of spatter generation during the laser–powder interaction are shown in Figure 5: (a) protrusion of the melt pool during LPBF under the combined effect of vapor recoil pressure and the Marangoni effect [53]; (b) formation mechanisms of spatter: three different types of spatters and typical spatter behavior during LPBF [77]. In order to understand the metal vaporization mechanism during the interaction between the laser and metal powder, more and more scholars use numerical simulation methods to analyze the melt pool, plume, and sputter formation process, showing the dynamic process of the laser and metal powder interaction, which gives us a more intuitive and comprehensive understanding of the metal vaporization process.

### 2.1. Numerical Simulation of Melt Pool Formation Process

**Figure 6:**
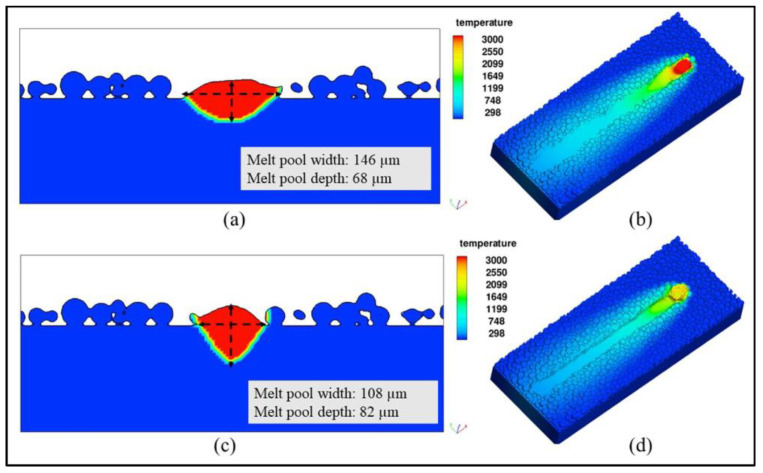
(**a**) The melt pool is wide and shallow when evaporation is ignored; (**b**) The melt pool temperature is overheated; (**c**) The melt pool is narrow and deep when combined with evaporation; (**d**) The maximum temperature is 2676 K when evaporation occurs [79].

**Figure 7:**
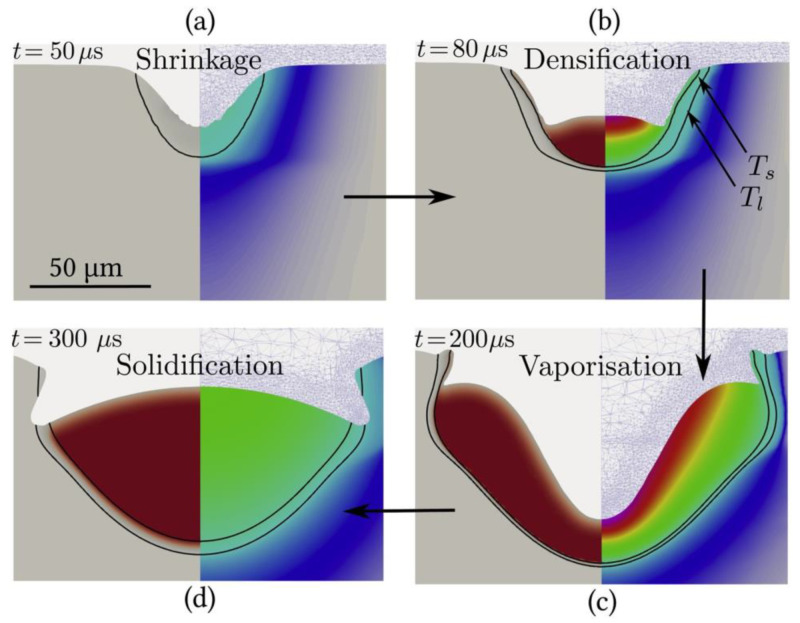
(**a**) Shrinkage of the powder; (**b**) Densification of the powder into a molten pool; (**c**) Vaporization phase with recoil pressure; (**d**) The onset of solidification [80].

**Figure 8:**
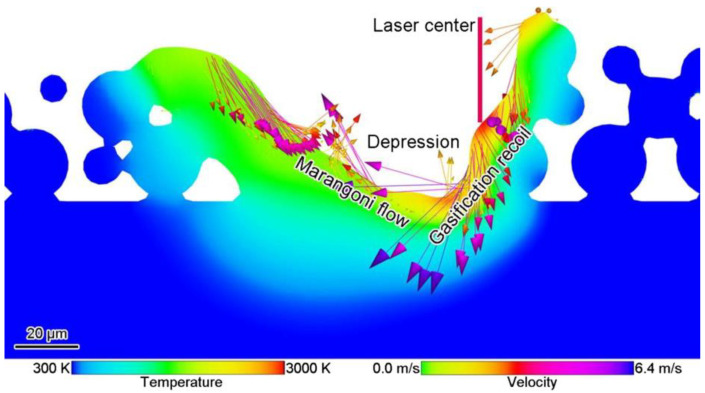
Melt pool dynamics including the Marangoni effect and gasification reaction [81].

**Figure 9:**
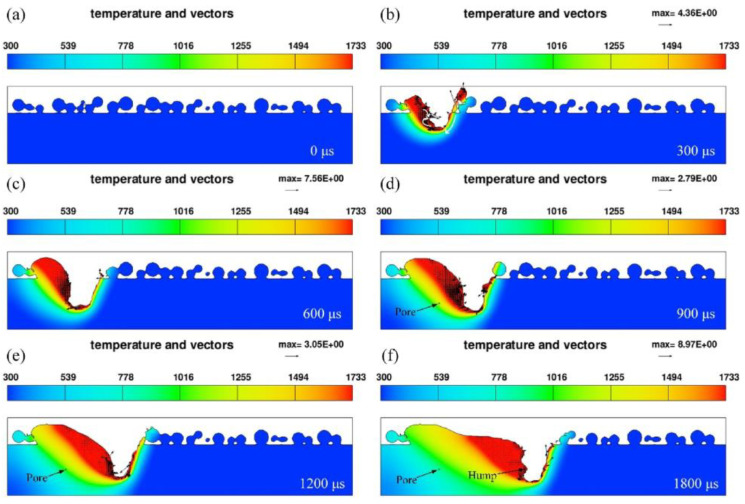
Pore formation process [83].

**Figure 10:**
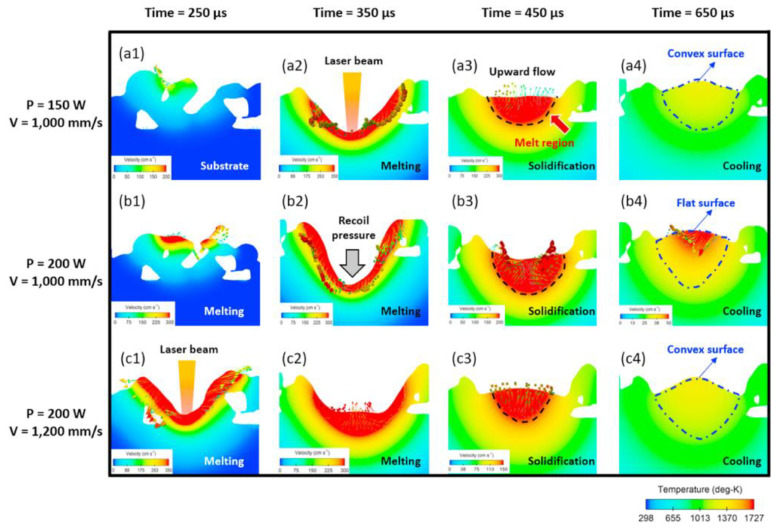
Variations of melt pool morphology under different process parameters [84].

**Figure 11:**
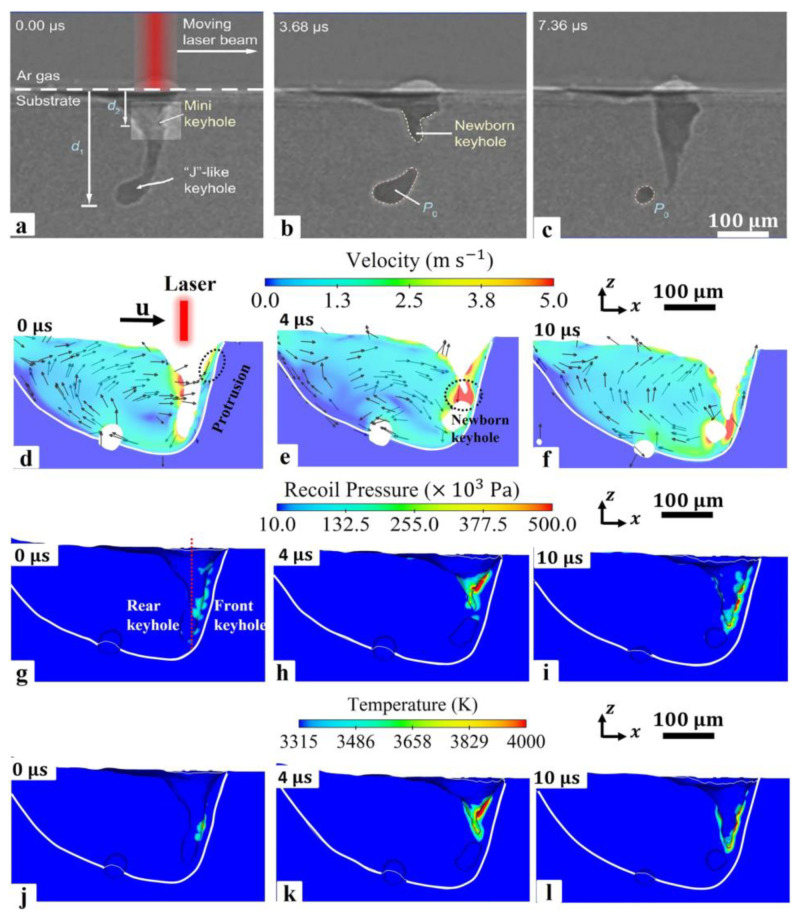
The instability of the keyhole leads to the formation process of stomata [85].

The melt pool is important in the LPBF process, and the size and morphology of the pool have a significant effect on part properties, including microstructure, hardness, and mechanical properties [61,78]. In the metal vaporization process, the molten pool is depressed by the recoil pressure of the vapor to form a keyhole. Yu-Che Wu et al. [79] used the discrete element method to numerically simulate and experimentally verify the melt pool behavior of the selected area laser melting process to numerically simulate whether the metal powder is evaporating and found that the melt pool is wide and shallow when evaporation is ignored and narrow and deep when evaporation is considered, as shown in Figure 6. Meanwhile, Alexis Queva et al. [80] simulated the successive stages of the laser and metal powder interaction process of the IN718 nickel-based high-temperature alloy using the level set finite element analysis method, as shown in Figure 7. The dynamic behavior of the melt pool process is also influenced by thermodynamic factors, such as the Marangoni effect, evaporation heat dissipation, etc., in addition to the vapor back pressure effect. Cao [81] developed a multi-physics factor model of the dynamic behavior of the melt pool based on the particle scale, as shown in Figure 8. During keyhole formation in the melt pool, rapid keyhole collapse can lead to the presence of inert gas in the solidified metal, which in turn leads to gas porosity formation [82]. Yunfu Tian et al. [83] performed a numerical simulation for experimental verification using single-track laser scanning; the experimental study showed that insufficient laser energy input made the melt pool and keyhole unstable and produce porosity defects, as shown in Figure 9. Laser power and process parameters are closely related, as a lack of laser power leads to melting pool instability and defects such as porosity. Patiparn Ninpetch et al. [84] studied the thermal behavior and molten metal flow characteristics by the discrete element method (DEM) and the computational fluid dynamics (CFD) numerical modeling method to analyze the influence of process parameters on the scanning orbit and obtained the evolution of the melt pool for different process parameters, as shown in Figure 10. Meanwhile, Lu Wang et al. [85] used a coupled multi-physical field model including heat transfer, liquid flow, metal vaporization, margin effect, and Darcy’s law for numerical simulation, mainly simulating the velocity field and temperature field of the melt pool, etc., while using high-speed X-ray imaging for experimental verification, and found that the uneven distribution of recoil pressure on the surface of the keyhole increases the formation of keyhole pores; in addition, different process parameters also affect the formation of keyholes, while low ambient pressure can reduce or even eliminate the formation of keyhole pores, and the instability of the keyhole leads to the formation process of pores, as shown in Figure 11. In addition to the numerical simulation analyses of the melt pool by the above scholars, other scholars have also conducted related studies [86,87,88,89,90,91,92,93], where scholars have mainly focused on the process of keyhole generation, the influence of process parameters and processing atmosphere on the melt pool morphology, and the relationship between the unstable state of the melt pool and part defects. At the same time, a multi-physics field coupling model was established to simulate the process of laser and metal powder interaction in a more realistic way. Therefore, the influence of metal vaporization on the melt pool morphology can be adjusted, and the melt pool morphology can be made more stable by optimizing the process parameters and changing the processing atmosphere, which in turn can produce a more stable keyhole morphology and reduce the generation of porosity defects.

### 2.2. Numerical Simulation of Plume and Splash Formation Process

**Figure 12:**
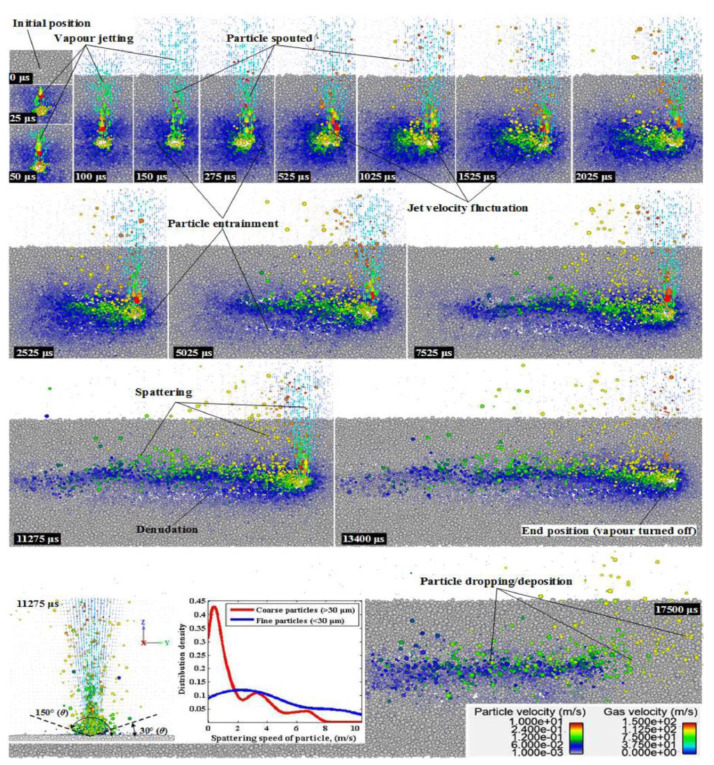
Multiphase flow simulation of powder particles and gas phase kinetic behavior [95].

**Figure 13:**
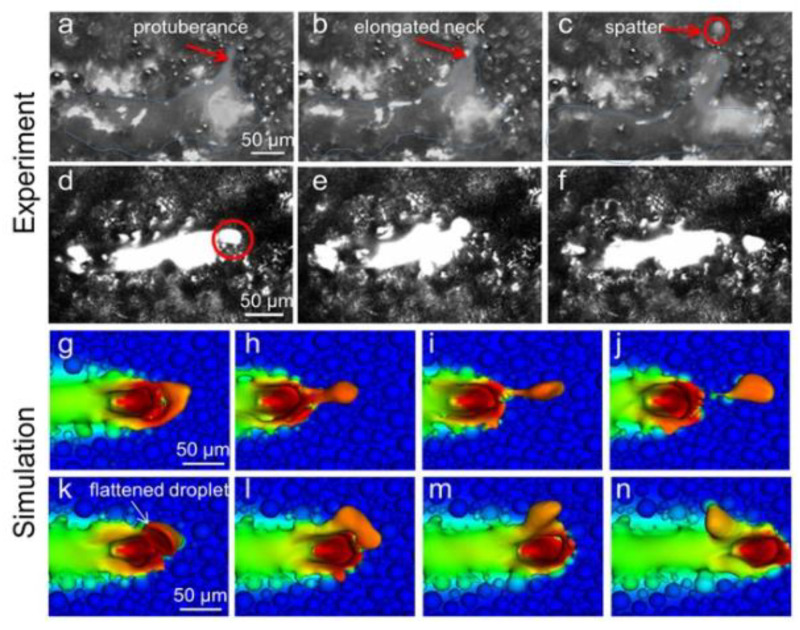
Simulation and experiment of droplet injection of the powder layer [66].

**Figure 14:**
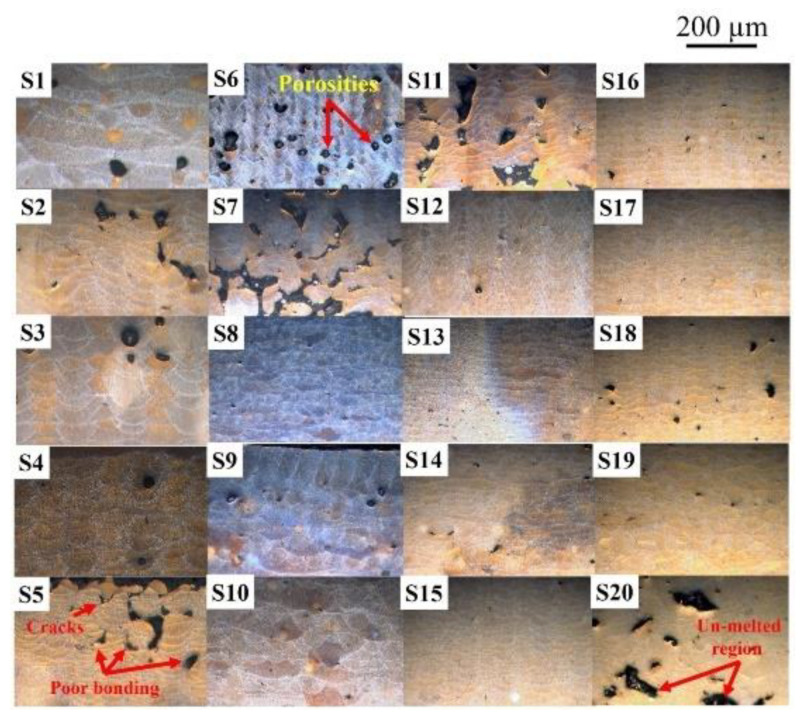
Splash defects [96].

**Figure 15:**
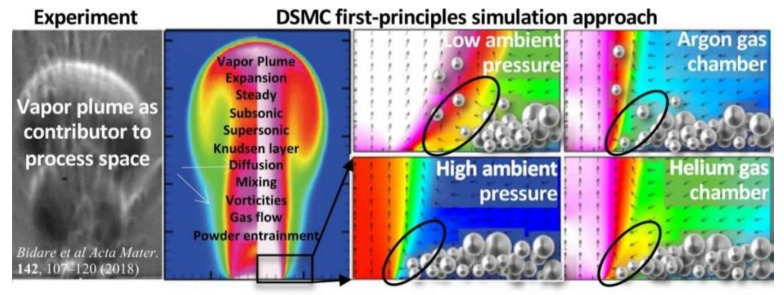
Plume high-speed schlieren imaging and numerical simulation map [68,97].

The metal vaporization process also produces plumes and splash vaporization products, and the formation of these vaporization by-products is a complex process that causes manufacturing defects in metal parts [94]. Therefore, in order to understand the evaporation process, most scholars use numerical simulations. Hui Chen et al. [95] established a multiphase flow model, which includes the momentum and energy exchange between powder particles and gas, by constructing a bidirectional coupled discrete element method and a finite volume method to lay the foundation for understanding the generation of sputtering and spalling during laser powder bed melting. This is the first time that the kinetic behavior of the gas phase and powder particles in the sputtering and spalling phenomena are simultaneously reproduced in numerical simulations, which were in good agreement with the experimental observations. It was also found that in the range of 60° to 120°, the jet angle has no significant effect on spalling and flaking, but when the jet angle is larger than 150°, the vortex flow behind the steam jet disappears and the radial expanding steam jet blows away most of the particles, resulting in the complete exposure of the spalling zone, the multiphase flow simulation of powder particles, the kinetic behavior of the gas phase, and the motion of the sputtering particles can be clearly seen in Figure 12. Sonny Ly et al. [66] also performed a multi-physics field coupled model for simulation and experimental validation to investigate the physical process effects associated with droplet spray generation, including the interactions between the metal powder particles and the surrounding gas dominated by the entrainment, vapor jet, and material injection processes, as shown in Figure 13. Sputtering can cause defects in parts. Asif Ur Rehman et al. [96] used a discrete element modeling approach to illustrate sputter formation and sputter-induced defects during LPBF of AlSi10Mg alloy, as shown in Figure 14. In addition to numerical simulations of sputtering, Michael A. Stokes et al. [97] conducted numerical simulations of stainless steel (SS316L) metallic material to study the physical processes of the vapor plume, as well as powder particle interactions, during LPBF, and the simulations captured the transition of the vapor plume structure from unsteady to steady state vapor flow; and at the same time, there was good agreement with the grain shadow images taken by high-speed grain shadow imaging, which helps us to better understand the laser–matter interaction process, as shown in Figure 15 for the plume high-speed grain shadow imaging and numerical simulations. The numerical simulation analyses of vapor plume and sputtering by scholars are mainly focused on studying the interaction between the reduced laser powder processing atmosphere and the multiphase coupling model, the principle of sputtering generation and the effect of sputtering on the formation of defects, and the effect of process parameters and processing atmosphere on vapor plume and sputtering by changing different process parameters and the concentration of argon in the processing atmosphere for simulation. In addition to the numerical simulations of the steam plume and splash by the above scholars, other scholars [98,99,100,101,102,103,104] also performed simulations using a coupled multi-physics field model to study the formation motion process of the steam plume and splash.

## 3. Visual Measurement Methods of the Molten Metal Evaporation Process in LPBF

Section 2 has discussed the metal vaporization mechanism, and many scholars have used numerical simulation methods for simulations to deepen our understanding. Next, visual measurement methods have been used to monitor and analyze the metal evaporation process, and then the process of laser and powder bed interaction has been controlled by optimizing the process parameters and changing the processing atmosphere to reduce the part defects and improve the part-forming quality and performance.

### 3.1. High-Speed X-ray Imaging

**Figure 16:**
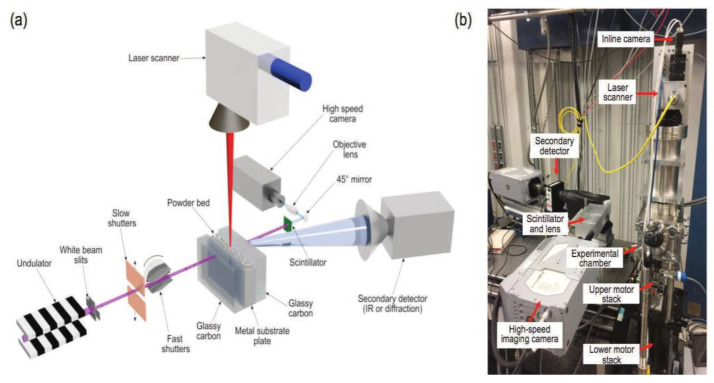
(**a**) Schematic of the X-ray experiment on laser powder bed fusion; (**b**) Photograph of the laser powder bed fusion simulator, along with the high-speed X-ray imaging detector and the complementary detection system [105].

**Figure 17:**
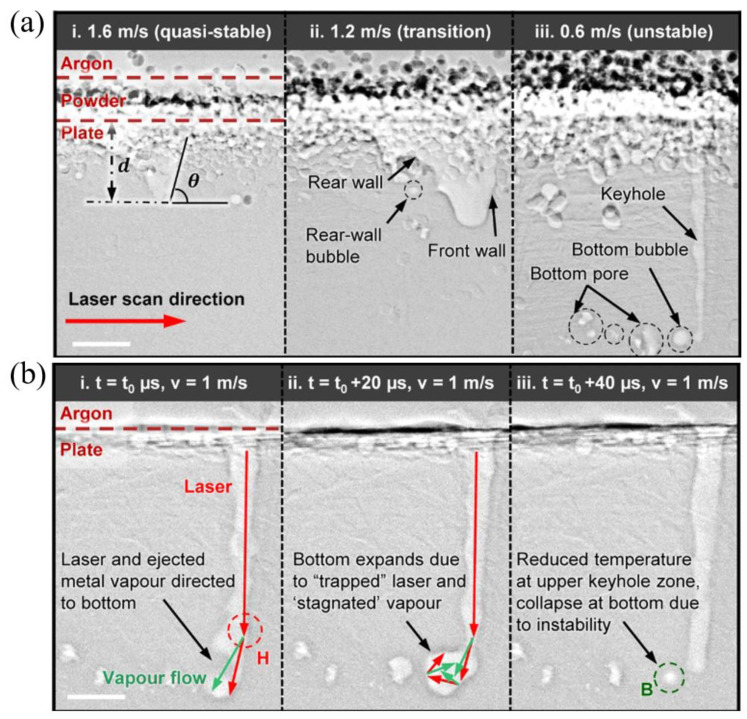
(**a**) Keyhole morphology variations from wide and shallow to narrow and deep through the (**i**) quasi-stable, (**ii**) transition, and (**iii**) unstable keyhole regimes under different laser scan velocities; (**b**) Keyhole collapse in the unstable state to form air holes [106].

**Figure 18:**
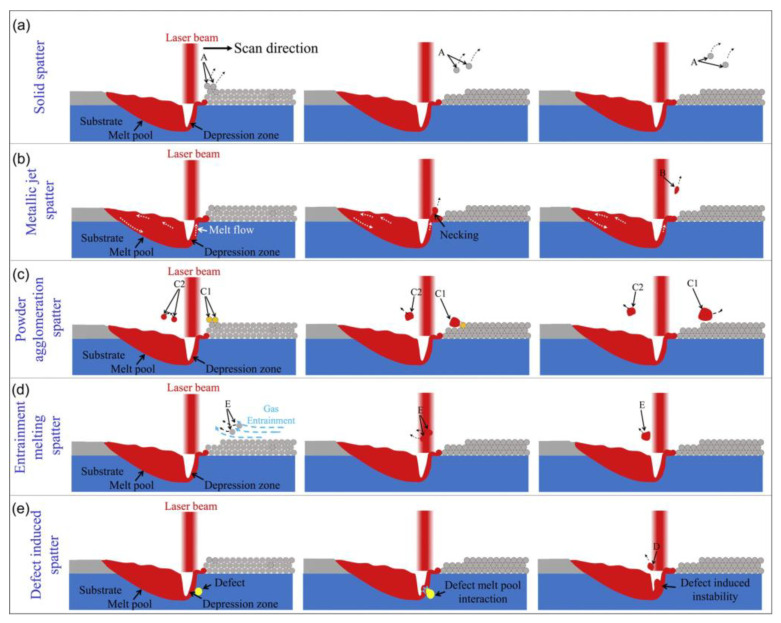
Schematic showing the formation mechanisms of all spatter types: (**a**) solid spatter (A); (**b**) metallic jet spatter (B); (**c**) powder agglomeration spatter (C1, liquid–solid agglomeration spatter; C2, liquid–liquid agglomeration spatter); (**d**) entrainment melting powder spatter (D); and (**e**) defect induced spatter (E) [108].

**Figure 19:**
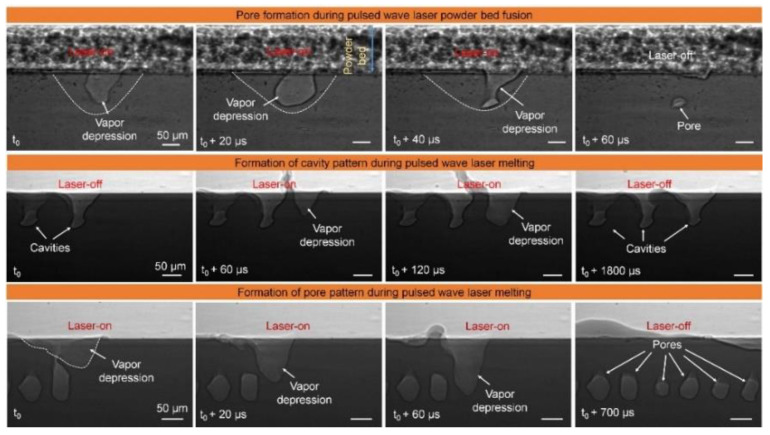
LPBF pore formation process [110].

**Figure 20:**
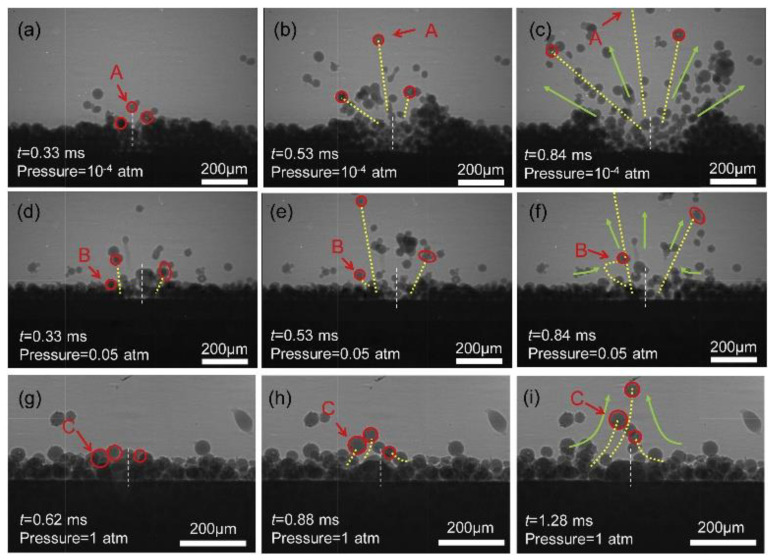
Dynamic X-ray images showing the movement of the powder at different times and pressures [111].

**Figure 21:**
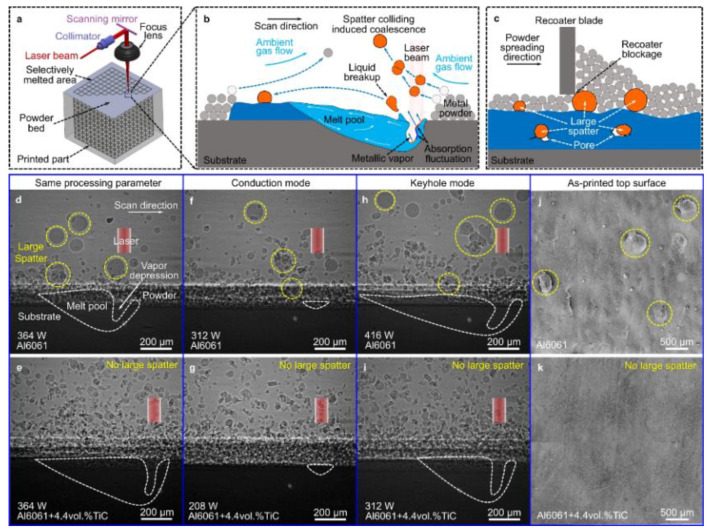
Before and after images of nanoparticle addition in the LPBF process [112].

The LPBF process is characterized by fast laser scanning speed, small melt pool size, and rapid melt pool solidification, and the vapor pressure and melt pool dynamics generated during metal vaporization occur within the melt pool. Recently, it has been discovered that a high-speed X-ray imaging technique, as shown in Figure 16, can be used to observe the dynamic microstructure and defect formation inside metal powders in real-time, such as the motion of the melt pool, due to its high spatial and temporal resolution and ability to penetrate metal powders, to study critical problems that could not be explored before, such as sputtering, keyhole formation, melt pool, and porosity [105]. First, Yuze Huang et al. [106] revealed keyhole and stomatal behavior using high-speed X-ray imaging to quantify the kinetics of their formation and found experimentally that keyhole pores are generated not only in the unstable case, but also in the transitional keyhole region generated at high power and scanning speed, while stomatal formation was observed, as shown in Figure 17. Ross Cunningham et al. [107] also investigated the keyhole during LPBF using ultrafast X-ray imaging, and the experimental results showed that the keyhole existed in the range of laser power and scanning speed used, and the transformation process followed the sequence of metal vaporization, melt pool liquid depression, and keyhole formation during high-energy laser irradiation of the metal using ultrafast X-rays; in addition to the keyhole, sputtering motion could also be observed. Zachary A. Youngdeng et al. [108] revealed the characteristics and formation mechanisms of five types of splashes in the LPBF process by high-speed in-situ X-ray imaging, the observed splashes were quantified by their velocity, size, and direction, the effects of laser power, scanning speed, and ambient pressure on the formation and characteristics of the five types of splashes in the LPBF process were investigated, and the characteristics and formation mechanisms of the five types of splashes in the LPBF process were shown as in Figure 18. In order to be able to eliminate the pores generated by stomata, Chu Lun Alex Leung et al. [109] investigated the underlying physical phenomena of laser–matter interaction between the first and second layers by in-situ high-speed X-ray imaging, showing that the steam jet promotes the formation of melt trajectories and exfoliation zones by sputtering, and also revealed the mechanisms of Marangoni-driven pore formation and pore dissolution and dispersion by laser remelting. S. Mohammad. H. Hojjatzadeh et al. [110] revealed the mechanism of pore formation during LPBF in real time using high-speed X-ray imaging, revealing that melt ejection and rapid melt pool solidification during pulsed laser melting led to the formation of the keyhole, and also revealed the kinetics of pore formation to provide guidance for the development of pore elimination methods; the pore formation process is shown in Figure 19. In addition, to reduce the effect of sputtering, Qilin Guo et al. [111] used in-situ high-speed X-ray imaging to observe the transient kinetic process of powder sputtering during LPBF and quantified the moving velocity, acceleration, and driving force of powder motion induced by metal vapor jet/plume and argon gas flow; the data quantified in this paper are important for developing accurate predictive powder sputtering models and can also be used to fit uncertainty constants and validate the model, which is important for developing new process techniques to reduce powder sputtering. Dynamic X-ray images showing the powder motion at different times and pressures are shown in Figure 20. Ming lei Qu et al. [112] showed that large spatters can be eliminated by using nanoparticles that can control laser–powder bed interaction instabilities, and verified this using in-situ high-speed X-ray imaging, ultimately finding two synergistic effects to prevent large spatter formation: (1) nanoparticle-enabled control of molten pool fluctuation eliminates the liquid-breakup-induced large spatters; and (2) nanoparticle-enabled control of the liquid droplet coalescence eliminates liquid-droplet-colliding-induced large spatters. The nanoparticles found in this paper simultaneously stabilize the melt pool fluctuation and prevent droplet aggregation, providing a method to eliminate large splashes in metal additive manufacturing, and the images before and after the addition of nanoparticles in the LPBF process are shown in Figure 21.

The scholars using X-ray mainly focus on the study of melt pool dynamics, sputtering dynamics, metal powder phase change process, etc.: for the melt pool, they mainly study the formation process of the keyhole and the stability of the melt pool; for the sputtering dynamics, they mainly study the formation process of sputtering, and the direction of motion, speed, quantity, etc.; for the metal powder phase change process, they mainly study the metal vaporization process, the resulting vapor plume, and recoil pressure. The X-ray effect allows us to observe the changes inside the metal powder during the interaction of the laser and the metal powder. The above studies help us to investigate the causes of the forming defects of parts and thus find ways to reduce the forming defects of parts. In addition to the above scholars who have used X-rays to study the process of laser interaction with metal powder, there are other scholars who have also conducted relevant studies using X-rays [113,114,115,116,117,118,119,120,121]. X-ray observation of the microstructure of molded parts has also been used to determine the internal grain boundary distribution [122] and to find microscopic defects (internal porosity, cracks, etc.). S. Mohammad H. Hojjatzadeh et al. [123] investigated the formation of porosity during LPBF using X-ray and multi-physics field simulation models and found that the high thermal capillary force generated by the high-temperature gradient in the laser action zone can rapidly eliminate porosity in the melt pool, providing guidance for achieving porosity-free 3D printing. Hossein Ghasemi-Tabasi et al. [124] also observed the crack formation process using X-rays in a miniaturized LPBF simulation chamber as a way to help us understand the mechanism of crack formation and provide guidance for the elimination of crack defects. The above study helps us to better understand the process of the interaction of the laser and the metal powder in the LPBF process as a way to improve the manufacturing quality and the performance of parts.

### 3.2. High-Speed Visible Light Camera Imaging

**Figure 22:**
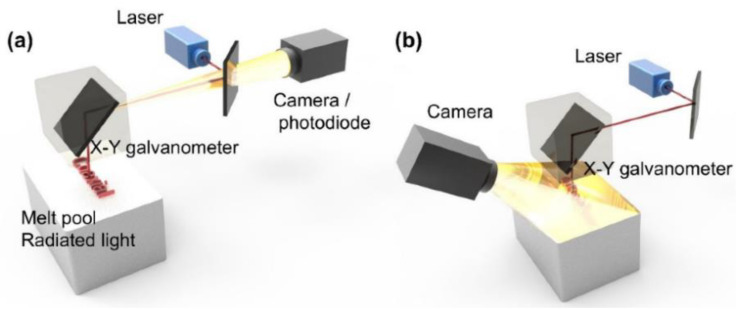
LPBF monitoring system: (**a**) coaxial (shared optical path with the laser) and (**b**) paraxial (at an angle to the laser) [23].

Due to their high frame rate and resolution, high-speed visible light cameras are widely used for online and offline inspection of LPBF. The obtained images can be enhanced, feature-extracted, and run through target recognition and target segmentation to extract the surface shape of the melt pool, plume, sputtering evaporation products, holes, areas without fusion, and other forming defects, and then optimize the process parameters and change the processing atmosphere to reduce defects and improve the forming quality and mechanical properties of parts. The coaxial system is the camera and laser optical path sharing a common optical path, and the side axis is the camera and laser optical path at a certain angle, as shown in Figure 22 [23]; the coaxial system is mainly used for inspection of the melt pool surface morphology, and the side-axis system is mainly used for sputtering inspection. In contrast, the side-axis system is more adaptable and is not limited by the original laser optical path.

**Figure 23:**
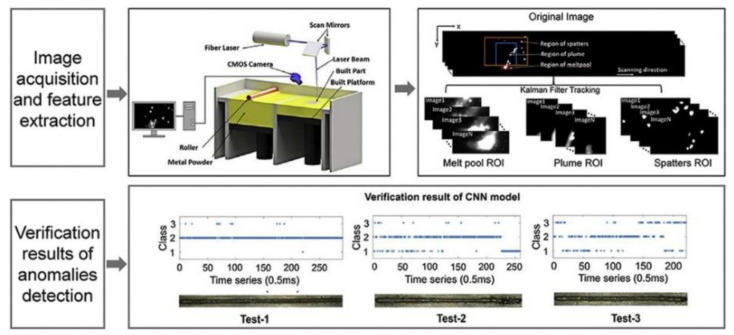
Setting up a high-speed visible side-axis monitoring system and extraction results [125].

**Figure 24:**
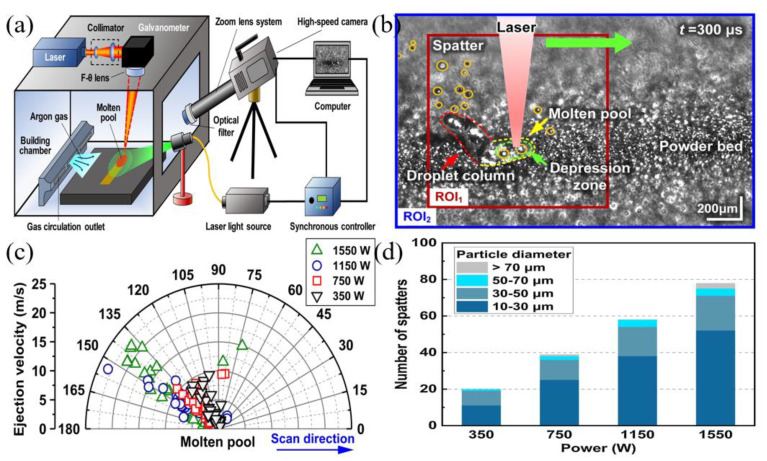
(**a**) Schematic diagram of the experimental setup for in-situ high-speed high-resolution imaging of LPBF; (**b**) Laser-matter interaction of chromium–nickel–iron alloy 718 powder bed LPBF; (**c**) Polar plots of the average jet velocity and the average jet angle of the spattered material at different laser powers; (**d**) The number and particle size of the spattered material as a function of laser power [126].

**Figure 25:**
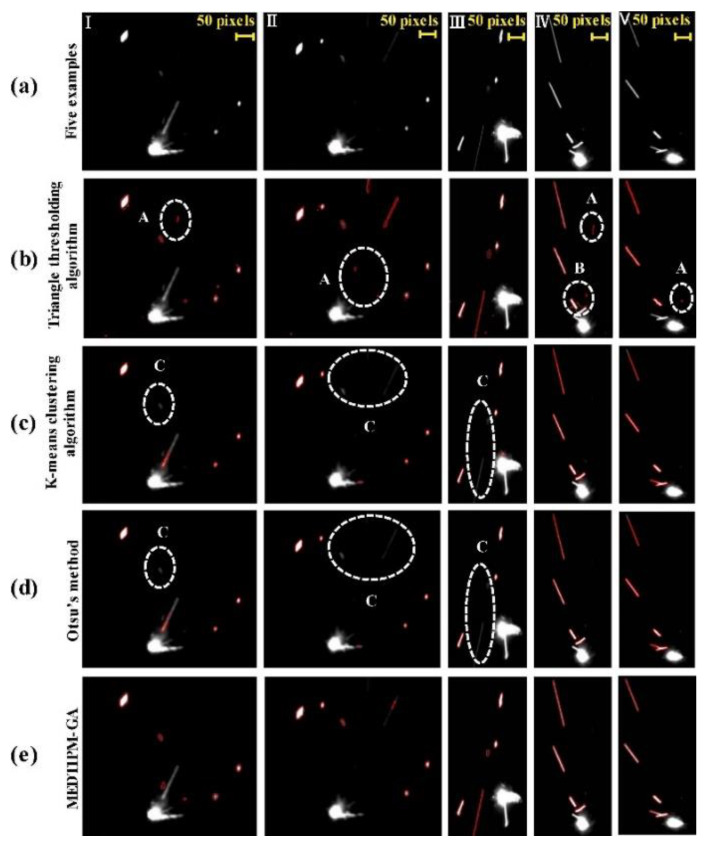
(**a**) High-speed camera image acquisition; (**b**) Triangle threshold segmentation algorithm; (**c**) K-means clustering algorithm; (**d**) Otsu’s method; (**e**) MEDTIA-GA [127].

**Figure 26:**
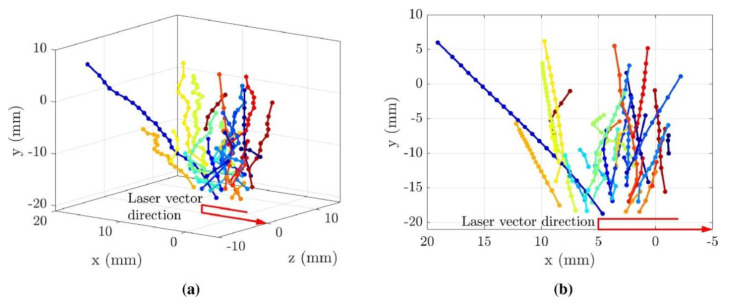
(**a**) Isometric view; (**b**) X-Y plane of particle tracks from the turnaround experiment (showing one in every three tracks for clarity, with different colors corresponding to different particles) [128].

**Figure 27:**
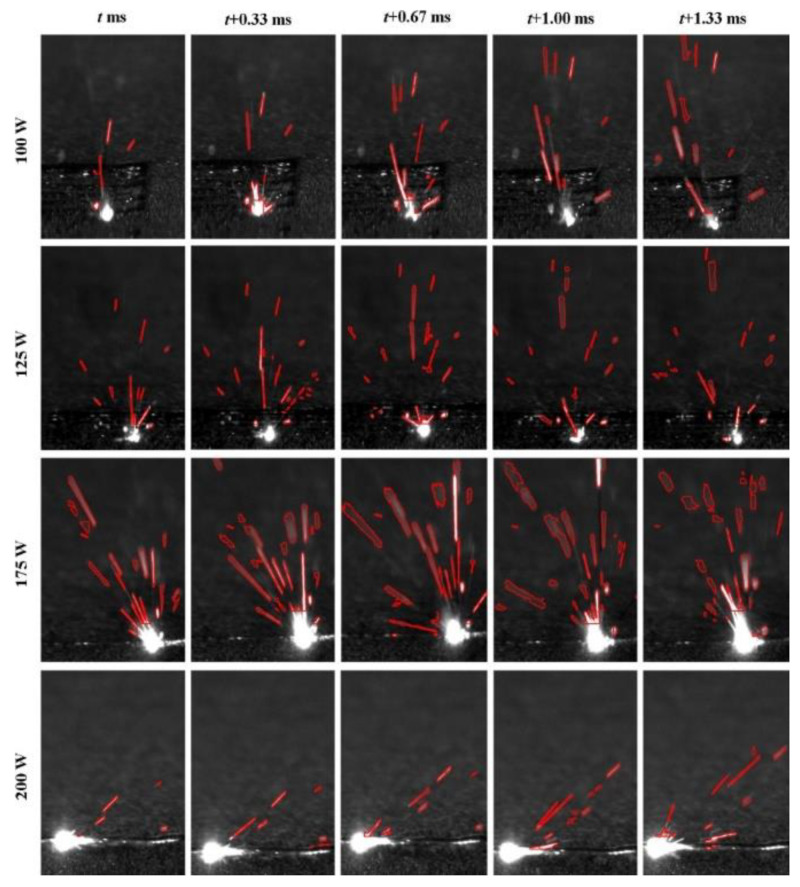
Spatter extraction results of the NN-based image segmentation method under four different laser powers [129].

High-speed visible light cameras can visually monitor the behavior of melt pools, splashes, vapor plumes, etc. They can also quickly identify defects, while methods such as image processing and machine learning enable the extraction of melt pool, splash, and plume features and their analysis. Yingjie Zhang et al. [125] used a high-speed visible light camera to build a side-axis monitoring system for image acquisition of the melt pool, plume, and splash during the melting process of the laser powder bed, and then used support vector machine (SVM) and convolutional neural network (CNN) methods to extract the melt pool, plume, and splash from the images, and found that the recognition accuracy of CNN was as high as 92.7%, which was higher than that of the recognition rate of the support vector machine (SVM); the results of setting up a high-speed visible side-axis monitoring system and the extracting results are shown in Figure 23. Jie Yin et al. [126] built a high-speed high-resolution imaging technique to study the laser–matter interaction in LPBF, and the melt pool, vapor plume, and droplet splash could be clearly observed by the image filtering algorithm and image enhancement method; the characterization analysis of the melt pool and splash showed that the melt pool characteristics and splash behavior depend on the laser input energy, the average ejection velocity and ejection angle of the splash increase with the laser power, and the high power laser tends to produce a large splash. The built side-axis shooting system and the observed images are shown in Figure 24. Dekun Yang et al. [127] built a side-axis monitoring system to capture splash images; proposed a genetic-algorithm-based maximum entropy double-threshold image processing algorithm to extract splash features in images; used the Otsu method, triangle threshold segmentation algorithm, and K-means clustering algorithm for comparison; and found that the maximum entropy double-threshold image processing algorithm can eliminate errors such as noise, splash adhesion, and splash omission. Finally, the relationship between splash area and splash number and laser energy density was analyzed; the paraxial shooting system built for LPBF and the extraction results are shown in Figure 25. Ralf D. Fischer et al. [128] built a high-speed all-optical camera to obtain the three-dimensional sputtering particle trajectories generated by the laser and powder interaction in LPBF and also calculated the sputtering particle velocities, which are important for predicting the sputtering particle’s landing point location on the powder bed and provides a new perspective for the subsequent analysis of the forming quality. The schematic diagram of the built light field camera and the three-dimensional sputtering and particle trajectories are shown in Figure 26. Zhenbiao Tan et al. [129] built a side-axis monitoring system to capture melt pool, plume, and splash images, proposed a segmentation method based on a CNN, which segmented the image into a block network, used a CNN and threshold neural network (TNN) to segment each block, and finally extracted 80.48% of the splash, while the splash connected to the melt pool could be extracted. The constructed lateral axis monitoring system and the extracted splash images are shown in Figure 27. Meanwhile, Heng Ma et al. [130] developed a single high-speed coaxial camera temperature measurement system for the laser powder bed melting process based on the dual-wavelength temperature measurement principle, proposed a dual-wavelength image matching method with sub-pixel accuracy and an overall parameter calibration optimization method, conducted experiments using the built experimental equipment, obtained single-line scan, single-layer scan, and multi-layer scan images of melt pool temperature field and melt pool morphology and visualized them, and finally built images of melt pool temperature with time and different size images of melt pool morphology. Scholars have used high-speed visible light imaging techniques mainly to extract melt pool surface morphology and vaporization products such as vapor plumes and splashes while combining traditional image segmentation algorithms and newly developed CNN algorithms in deep learning to extract features in the images, which has laid the foundation for subsequent research on methods to improve part imaging quality and control part performance. Melt pool, vapor plume, and splash features are currently being studied using high-speed visible cameras, and there is further research on melt pool [131,132,133] and splash [134,135,136,137,138,139,140,141,142,143,144,145] characteristics.

### 3.3. High-Speed Schlieren Imaging

**Figure 28:**
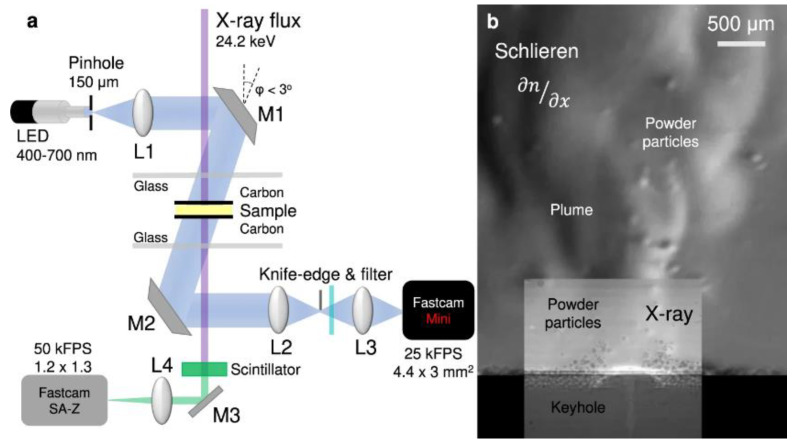
(**a**) High-speed grain shadow and X-ray device; (**b**) Composite image [76].

**Figure 29:**
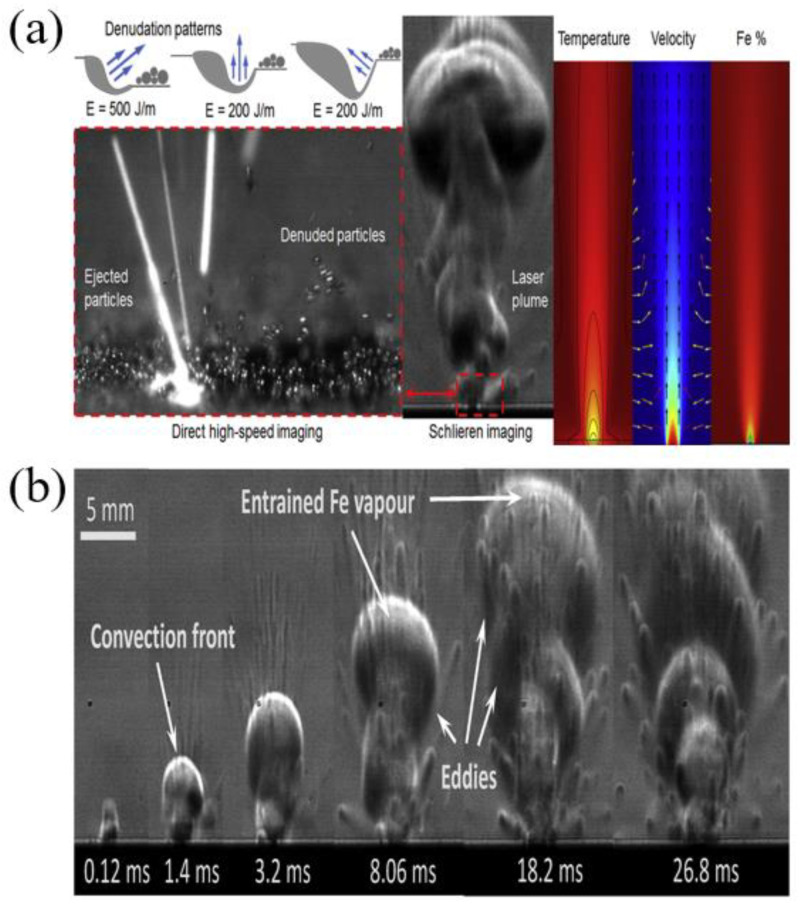
High-speed imaging and schlieren imaging: (**a**) high-speed imaging of the plume, schlieren imaging, and temperature field simulation images and (**b**) single-track laser scanning images of plume morphology changes [68].

**Figure 30:**
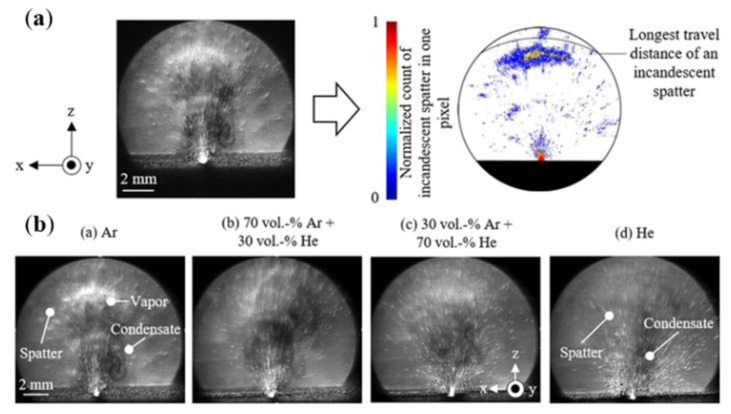
High-speed schlieren images: (**a**) splash extraction by thresholding and (**b**) schlieren images under different inert gas environments [154].

To visualize nonconstant gas flows with discontinuities, high-speed schlieren imaging techniques are often used [146]. High-speed schlieren imaging has been widely used to visualize gas flow in various applications, such as automotive aerodynamics, ballistics, and laser welding [147]. LPBF is a process in which metal vaporization occurs during the interaction between the laser and the metal powder. However, since the vaporization process cannot be observed with the naked eye, it is necessary to study it to help us understand the metal vaporization process and determine whether the process parameters are reasonable and the formation of vaporization products, such as vapor plume and sputtering by the vaporization phenomenon, is within acceptable parameters. Therefore, high-speed schlieren imaging is introduced to observe the process of evaporating metal. Deep learning has also been applied to image analysis of schlieren imaging systems, where neural networks can effectively capture flow structure features, such as excitation and vortices [148,149,150], and extract data information about the flow that can also be used for prediction [151] and reconstruction [152,153]. To understand how the melt pool and vapor plume interact during the laser and powder interaction, I. Bithara et al. [76] coupled the melt pool and plume dynamics by combining the high-speed schlieren imaging technique and in-situ X-ray method to correlate the vapor plume generated by the interaction of the laser and metal powder with the keyhole it creates in the melt pool, and judged the stability of the melt pool by the morphology of the vapor plume. The high-speed visualization of the fluid motion of the LPBF process helps us to design the process window with higher efficiency and speed, and lays the foundation for LPBF process monitoring with the combined imaging of high-speed schlieren imaging technology and X-ray imaging technology, as shown in Figure 28. Meanwhile, P. Bidare et al. [68] used a combination of high-speed imaging and schlieren imaging, as well as Multiphysics field simulations, to reveal the process of laser and metal powder interaction during LPBF. The numerical simulations also help us to understand and quantify the observed flow behavior by varying the process parameters, such as laser power and scanning speed, to observe the changes in the vapor plume morphology, which facilitates the characterization of hydrodynamic phenomena in the LPBF process, helping to prevent defects in additively manufactured parts. Figure 29 shows the usage of high-speed imaging and schlieren imaging. For vaporization by-products such as plumes and spatters, which can be effectively removed by changing the processing atmosphere, Siegfried Baehr et al. [154] studied the effect of different argon–helium gas mixtures compared to pure argon on by-products during the processing of high-strength aluminum alloys using high-speed grain shadowing, which allows visualization of by-products during the process, and then studied the evaporation phenomenon during the melting process of the laser powder bed. The images taken by high-speed grain shadowing are shown in Figure 30. Additionally, P. Bidare et al. [155] studied the state of the laser beam and powder plume in different processing atmospheres using high-speed imaging and schlieren imaging techniques. Scholars have now used high-speed schlieren imaging technology to study the interaction between laser and metal powder during the melting process of the laser powder bed, combining imaging analysis of melt pool and vapor plume dynamics, which helps us understand the process in a deeper way, and then correlate process parameters such as laser power, scanning speed, and processing environment with part performance and forming quality, helping us optimize process parameters at high efficiency and speed. However, there are still relatively few studies using a high-speed schlieren imaging system to study the melting process of the laser powder bed. With the rapid development of machine learning and artificial intelligence, it is an important trend to introduce deep learning and other methods into image analysis in high-speed schlieren imaging, which can help us to quantify the metal evaporation process by extracting the feature information in the image.

## 4. LPBF Process Inhibition of Metal Evaporation Measures

Through the numerical simulations of the coupled multi-physical field models of the LPBF process in Section 2 and Section 3 and the summary of the visual measurement methods by high-speed X-ray, high-speed visible light camera, and high-speed schlieren imaging techniques, it has been found that LPBF metal evaporation is a complex and highly dynamic process. Most researchers have mainly focused on the study of molten pools, vapor plumes, and sputtering. It has also been found that laser energy input and processing atmosphere are the main influencing factors of forming quality and defects in the metal evaporation process. Therefore, the suppression of evaporation can be considered in terms of laser energy input and processing atmosphere.

### 4.1. Laser Energy Density

The laser and metal powder interaction processes due to the different material properties of different materials, and therefore materials require different laser energy densities; meanwhile, the same material in different combinations of laser power and scanning speed parameters will produce different metal vaporization phenomena and vapor pressures on the surface of the melt pool, resulting in different keyhole morphologies. The laser energy density formula is [40]:

(1) $$E=\frac{P}{v\cdot h\cdot t}$$

**Figure 31:**
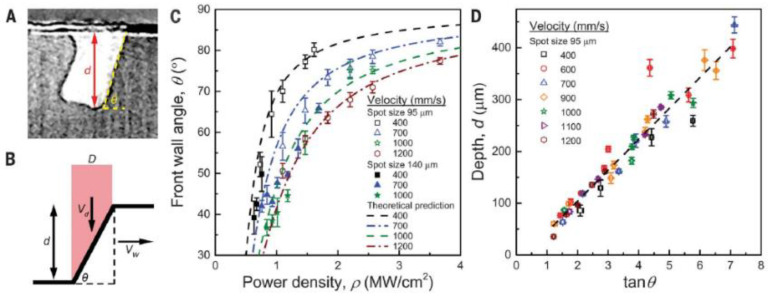
Relationship between keyhole depth, front wall angle, and laser energy density [107].

**Figure 32:**
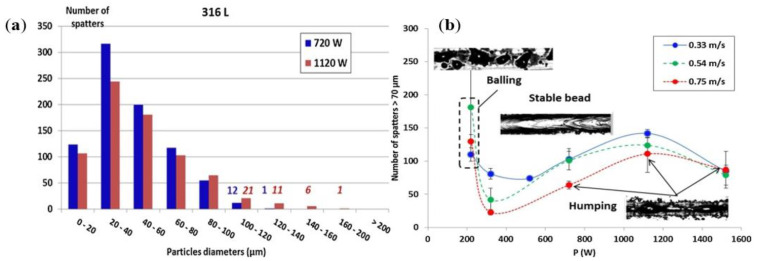
The number of spatters under different process parameters: (**a**) the number and size of spatters under different laser powers and (**b**) the effect of different lasers power and scanning speeds on spatters [156].

**Figure 33:**
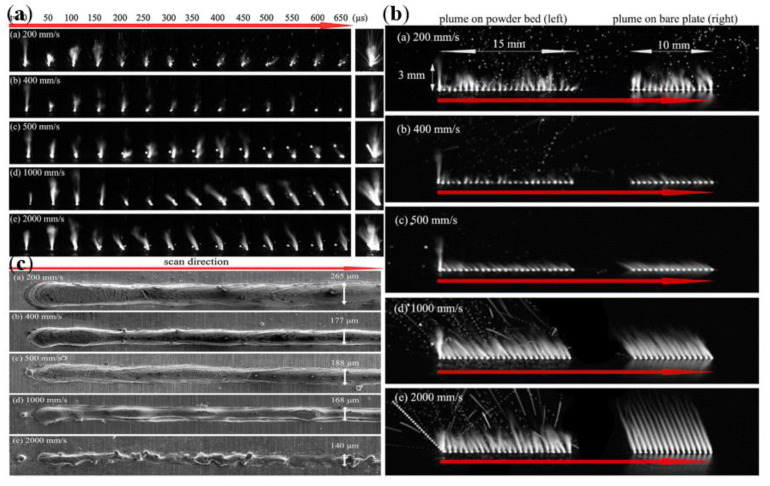
Vapor plume and spray images at different laser scanning speeds: (**a**) evolution of vapor plume and spray generation at different laser scanning speeds; (**b**) superimposed images of plume and spray at different laser scanning speeds; and (**c**) powder bed morphology at different scanning speeds [157].

In this equation, E is laser energy density (in J m^−3^), P is the laser power (in J s^−1^), v is the laser scanning speed (in m s^−1^), h is the hatch spacing (in m), and t is the powder layer thickness (in m); the above-influencing factors in the laser power and laser scanning speed are the main influencing factors. Ross Cunningham et al. [107] found a clear threshold from the conduction mode to the keyhole by high-speed X-ray imaging and established the relationship between the keyhole front wall angle, keyhole depth, and laser energy density, thus clearly finding that the formation of the keyhole in the melt pool is affected by the laser energy density, and the variation of the keyhole affects the stability of the melt pool and thus the forming quality. The relationship between the keyhole depth, front wall angle, and laser energy density is shown in Figure 31. V. Gunenthiram et al. [156] built a high-speed camera monitoring system to take spatter images of 316L stainless steel, experimented by varying the laser power and scanning speed in combination with different process parameters, and found that the different process parameters, such as laser power and scanning speed, lead to different laser energy densities, and thus the number and size of spatters produced is also different. The number of spatters with different process parameters is shown in Figure 32. Hang Zheng et al. [157] used high-speed visible imaging to build a paraxial monitoring system to observe the vapor plume and spatter generation process during single-pass forming of 304 stainless steel; the formation and evolution of the plume at different laser scanning speeds were observed in an attempt to establish the relationship between scanning speed, plume stability, spatter generation, and melt morphology. It was concluded that in the lower laser scanning speed range, the high laser energy density makes the vapor plume violently unstable and the high recoil pressure ejects the droplets from the melt pool; when the laser scanning speed exceeds a certain threshold, the vapor plume tilts backward with respect to the scanning direction. The images of the vapor plume and splash at different laser scanning speeds are shown in Figure 33. Through the above studies, it is found that the laser power and laser scanning speed in the laser energy density are the main factors affecting the LPBF process, and too large or too small a laser energy density is detrimental to the molten pool; therefore, scholars should keep studying this to find the appropriate laser energy density threshold for processing parameters to suppress the metal vaporization process, and other scholars have also conducted related studies on the influence of laser power and scanning speed on the metal vaporization process [158,159,160,161,162,163].

### 4.2. Processing Atmosphere

**Figure 34:**
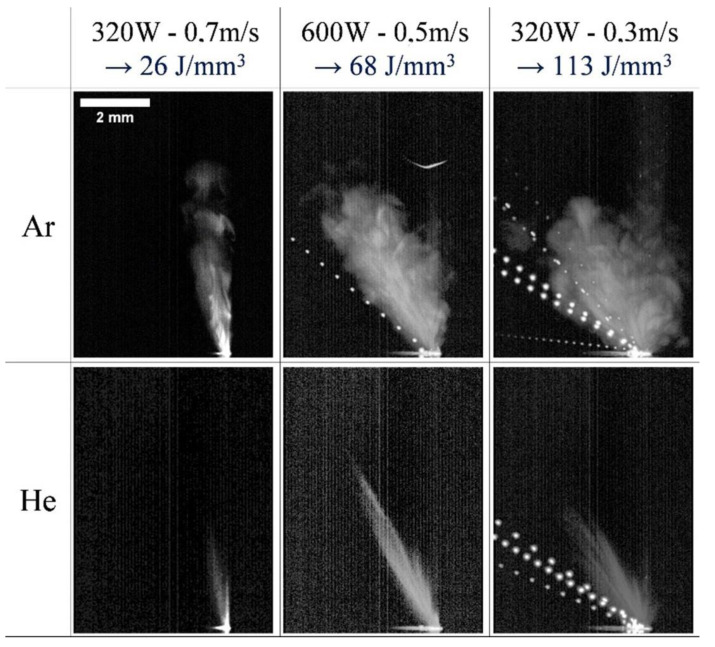
Overlay of 300 images from video recorded at 10,000 fps (bead-on-plate tests) showing vapor plume expansion under Ar and He [166].

**Figure 35:**
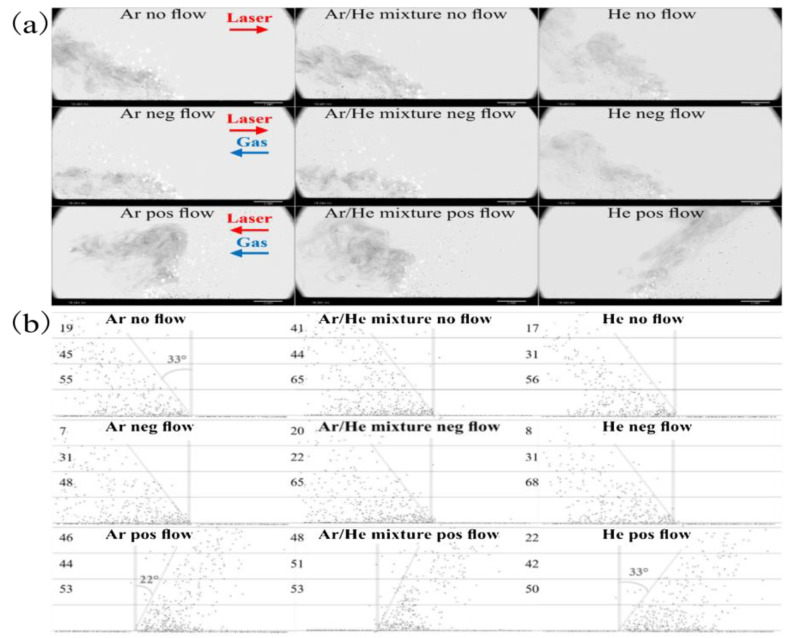
(**a**) Shadowgraphs extracted at 18,264 ms from the experiments performed under argon, the gas mixture, and helium for the no flow, negative flow, and positive flow configurations; (**b**) Analysis of the darker (colder) spatters from extracted shadowgraph [167].

**Figure 36:**
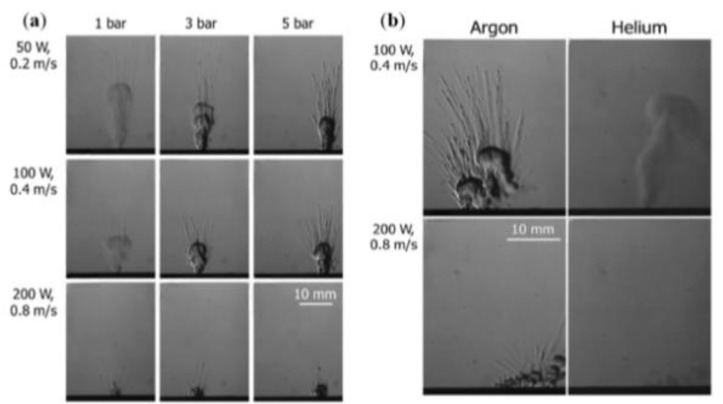
Plume images were observed using the high-speed schlieren technique: (**a**) Ripple images at different laser power, scanning speed, and pressure; (**b**) Images at different laser power, scanning speed, and inert gas [155].

This can be achieved by optimizing process parameters to achieve an appropriate laser energy density threshold, in addition to introducing recirculated gas streams of argon and other noble gases necessary to eliminate evaporation byproducts generated during metal evaporation [29]. Reducing the impurities that gradually fall on the protective mirror, so that the metal powder can fully absorb the laser energy, especially for metals with a high vaporization tendency, such as Zn and Mg [164,165], and also protects the metal from high-temperature oxidation, which plays a key role in the quality of the formed part. The influence of the current processing atmosphere on the metal vaporization process is as follows: S. Traore et al. [166] investigated the effect of the processing atmosphere on metal vaporization during LPBF, established the processing atmospheres of argon and helium, observed the state of the melt pool, vapor plume, and sputtering during LPBF of nickel-based alloys using high-speed visible imaging, and found that changing the gas atmosphere from argon to helium can affect the melt pool and vapor plume morphology as shown in Figure 34. C. Pauzon et al. [167] used high-speed shadowing imaging to image the LPBF process in the presence of pure argon, pure helium, and a mixture of argon and helium and found that pure helium reduced spatter by at least 60% and a mixture of argon and helium reduced spatter by 30% compared to pure argon. This high-speed shadowing demonstrates the accelerated expansion of the vapor plume with the addition of helium and the reduction in spatter and vapor accumulation at the laser spot, with images taken by high-speed shadowing as shown in Figure 35. P. Bidare et al. [155] studied the variation of vapor plume morphologies in the environment of two gases, argon and helium, and the same gas at different pressures using a high-speed visible light camera and high-speed schlieren imaging, from which the plume and spatter generated during the interaction between the laser and the metal powder can be clearly observed, as shown in Figure 36. From the above research, scholars found that with different inert gases in the same laser energy density value of the plume, spatter morphology is different, which may be due to the different physical properties of different inert gases (such as gas density, thermal conductivity, and other parameters): helium thermal conductivity is much larger than that of argon and nitrogen, so it affects the laser and powder in the process of heat transfer, and thus affects the morphology of the melt pool, plume, and spatter; with the same inert gas in the case of a different laser energy density threshold, plume and splash forms are also different due to a number of factors, such as vapor recoil pressure, the Marangoni effect, surface tension, etc. For the laser energy density threshold, the laser power and laser scanning speed are the main influencing factors: when the laser power and scanning speed are different, the dominant effects of steam recoil pressure, Marangoni effect and surface tension on the molten pool are also different. In summary, the processing atmosphere affects the metal evaporation process, but also shows that the LPBF process is a dynamic and complex process, which requires specific analysis of the metal evaporation process according to different process parameter conditions. However, by reading the literature, we found that there is little information about the influence of the wind speed of the inert gas entering the forming room from the air inlet on the metal vaporization process. Therefore, the study of the influence of the wind speed of the inert gas entering the forming room on the metal evaporation process may also become a hot research topic, because on one hand, different wind speeds of inert gas entering the forming room have different effects on the melt pool, and on the other hand, it can blow away the splashes and prevent them from falling into the printed part area, causing defects. Ultimately, a properly controlled machining atmosphere can reduce part defects and improve the quality of part forming. Other scholars have also conducted related studies on the effect of the processing atmosphere on the metal vaporization process [168,169,170,171,172,173,174].

## 5. Conclusions

LPBF is an evolutionary process involving multiple physical fields and complex dynamics to achieve high-performance and efficient part manufacturing. Macroscopic defects, such as cracks and warping, and microscopic defects, such as porosity and inclusions, can occur in the process. Therefore, the part manufacturing quality and performance are the key issues hindering the wide application of LPBF technology. Metal vaporization plays a key role in the quality and performance of part forming in LPBF. In recent years, metal vaporization and its effects have received increasing attention from scholars, especially for Zn, Mg, and other elements and their alloys that are prone to vaporization but also have important applications. This paper summarizes the current research status and future development direction of metal vaporization in the laser powder bed melting process.

(1) For the LPBF process, due to the metal vaporization process, vapor plumes, powder exfoliation, sputtering, and keyholes will be generated, and these phenomena can be visually observed by high-speed X-ray imaging technology, high-speed visible light camera imaging technology, and high-speed schlieren system imaging technology to understand the process of metal vaporization. The process of metal vaporization and the formation of the keyhole are caused by high temperatures in the melt pool due to the laser energy density input, and the laser energy density plays a dominant role in the formation of the keyhole, so the appropriate laser energy density is critical to the quality of forming. High-speed imaging technology can capture images of the melt pool surface morphology, the movement of sputtered particles and forming defects on the part surface, and the quality of the squeegee powder, etc., which helps us analyze whether the process parameters are set reasonably and facilitates narrowing the process window with high efficiency and speed.
(2) The interaction of a high-energy laser and metal powder is a complex dynamic process, which is accompanied by changes in mass, energy, and momentum during the melting of the metal powder, and also involves the influence of vapor recoil pressure, the Marangoni effect, surface tension, and other related forces on its vaporization process; however, this cannot be observed by the naked eye, and the establishment of a multi-physics coupled model can show more information about the forces involved in the vaporization process. The numerical simulations are necessary to help us visualize the vaporization process of the metal powder melting. The current numerical simulations mainly focus on the variation processes of the melt pool, and there are fewer studies on the evaporation products, such as plumes, sputters, etc. It is important to select an appropriate evaporation model for the numerical simulation of the LPBF process.
(3) The laser energy density, powder layer thickness, processing environment, and material properties are the main factors influencing the LPBF metal vaporization process. The evaporation of key metal elements has a critical influence on powder stripping, plumes, sputtering, porosity, incomplete fusion, and the segregation of alloy element composition. Therefore, the LPBF process requires an appropriate laser energy density threshold and an efficient gas recirculation system to suppress the metal vaporization process, maintain a stable melt pool during the laser and metal powder interaction, and perform with a stable melt trajectory to improve part imaging quality.

In future research, the following areas should be the main focus:

(1) To further explore the complex processes of the high-energy laser and metal powder in the laser powder bed process, it is important to understand the metal vaporization process and its effects on the LPBF process. A multi-physics field-coupled numerical simulation model is established, while the metal vaporization process is visualized using visual measurement methods, such as ultra-high-speed X-rays, high-speed visible light cameras, and high-speed Schlieren imaging systems. Dynamic information about melt pool temperature, melt pool morphology, keyhole evolution, powder motion, plume morphology change, sputter motion, and forming defects are obtained by the above methods to understand the metal evaporation process in depth. The effects of material properties, powder layer thickness, and processing conditions on the quality and performance of LPBF forming are considered from the perspective of metal evaporation, while sputtering is regulated by new materials, such as nanoparticles.
(2) Research on the generation of melt pools and evaporation by-products in the LPBF process, mainly through some new technical means, such as ultra-high-speed X-rays, can detect the internal changes in the process of laser and metal powder interaction, and a high-speed schlieren system can visualize the metal evaporation process by combining the melt pool images and evaporation product images for joint analysis, helping to reveal the metal evaporation process at a deep level and promoting the high-fidelity development of numerical simulations. By considering the effects of vapor recoil pressure, the Marangoni effect, and evaporation heat dissipation in the numerical simulation process, an accurate multi-physics coupled evaporation model can be established, which can provide a realistic simulation of the LPBF metal evaporation process and more accurately reproduce the laser and metal powder interaction process. However, numerical simulation is very computationally demanding and consumes computer resources. Therefore, multi-scale modeling will be needed in the future to improve computational accuracy and efficiency while revealing the interactions between materials, processes, structures, and properties with computational accuracy.
(3) Scholars should further explore the vaporization process of Zn, Mg, Al, and other metals and their alloy materials, especially focusing on increasing the research on Mg metals and their alloys. With the lowest density, high specific strength, biodegradability, and improved metabolism, Mg is widely used in aerospace, biomedical, automotive, and other fields, and has a wide range of development prospects. Mg loss due to low melting point/high saturation vapor pressure element vaporization is severe, resulting in alloy composition segregation and reduced part forming quality. To accurately control the composition and properties of LPBF parts, the metal powder material and process parameters should be adjusted and optimized to reduce vaporization loss. At the same time, the prediction of metal evaporation loss by numerical simulation should be further improved.
(4) The metal vaporization process is an important phenomenon in the process of laser and metal powder interaction, and it provides a variety of information for in-situ monitoring of the LPBF process, including melt pool, plume, and sputtering characteristics. This information includes acoustic, optical, thermal, and force signals; it is a key issue to extract the useful signals we need for quality monitoring and control, while the combined use of monitoring equipment, such as high-speed X-rays, high-speed visible cameras, pyrometers, thermal imagers, infrared cameras, and acceleration sensors, can provide even richer information. The use of artificial intelligence techniques such as machine learning (supervised, semi-supervised, and unsupervised) and computer vision to extract useful feature signals from LPBF process data for the analysis of metal evaporation processes is a major research trend.

The potential limitations or challenges:

High-speed X-ray imaging technology, high-speed visible light cameras, and high-speed schlieren imaging technology have been applied to the LPBF process, but there are some limitations and challenges. The two main areas include the following: the first is the issue of cost. Since the LPBF process has a fast laser scanning speed, large melt pool size, and other characteristics, ordinary industrial cameras cannot shoot the surface morphology of the melt pool, plume, and spatter; only with high frame rate, high-resolution, high-speed cameras can these details be observed, while only through high-speed X-ray imaging technology can we observe the internal morphology of the melt pool and the internal microstructure of metal powder. Furthermore, the observation of the vaporization process using high-speed schlieren imaging technology requires the use of a schlieren concave mirror. High-speed cameras, X-rays, and schlieren concave mirrors are all relatively expensive; The second is the optical path problem: high-speed X-ray imaging technology and high-speed schlieren imaging technology have high requirements for the optical path, requiring a special optical platform for the adjustment of the optical path, while high-speed camera imaging technology also requires setting the installation position of the camera and selecting the appropriate light source, etc. These are the difficulties faced in achieving accurate measurements of the metal vaporization process.

## Data Availability

Not applicable.
